# Evaluation of the Effect of *Pistacia atlantica* Oleoresin on Blood Sugar, Pressure and Lipids in Patients With Type 2 Diabetes: A Single‐Blind, Placebo‐Controlled Trial

**DOI:** 10.1002/edm2.504

**Published:** 2024-06-16

**Authors:** Zahra Memariani, Mahin Tatari, Maryam Zahedi, Zahra Hesari, Ali Davarian, Fatemeh Kolangi

**Affiliations:** ^1^ Pharmaceutical Sciences Research Center Babol University of Medical Sciences Babol Iran; ^2^ Traditional Medicine and History of Medical Sciences Research Center, Health Research Institute Babol University of Medical Sciences Babol Iran; ^3^ Statistical Science Department Sapienza University of Rome Rome Italy; ^4^ Clinical Research Development Unit (CRDU) Golestan University of Medical Sciences Gorgan Iran; ^5^ Laboratory Sciences Research Center Golestan University of Medical Sciences Gorgan Iran; ^6^ Ischemic Disorders Research Center Golestan University of Medical Science Gorgan Iran; ^7^ Counseling and Reproductive Health Research Centre, Department of Persian Medicine, School of Medicine Golestan University of Medical Sciences Gorgan Iran; ^8^ Food, Drug, Natural Products Health Research Centre GolestanUniversity of Medical Sciences Gorgan Iran

**Keywords:** diabetes, integrative medicine, medicinal herbs, Persian medicine, *Pistacia*

## Abstract

**Background:**

Diabetes mellitus (DM) is a chronic metabolic disorder characterised by high blood sugar (BS) levels due to impaired insulin production or insulin resistance. It is a global health concern with significant implications for morbidity and mortality. Persian medicine has long utilised natural remedies, such as *Pistacia atlantica* Desf., for various diseases. In this randomised clinical trial, the effects of *P. atlantica* oleoresin in the improvement of lipid profiles, glucose indices and blood pressure (BP) were assessed in patients with Type 2 DM.

**Materials and Methods:**

In this randomised, single‐blind, placebo‐controlled study, 42 patients with Type 2 DM were randomly allocated to receive either *P. atlantica* oleoresin or placebo capsule for 3 months. Patients were evaluated prior to and 12 weeks after the beginning of the intervention, in terms of changes in lipid profiles, glucose indices and BP.

**Results:**

After 3 months, the mean BP in patients with DM receiving *P. atlantica* oleoresin was significantly reduced compared with the baseline (*p* = 0.001). Also, these changes were significantly higher than those of the control group. The mean of total cholesterol (*p* = 0.89), low‐density lipoprotein (LDL) (*p* = 0.43) and triglyceride (TG) (*p* = 0.98) in the intervention group after 3 months was lower than that in the control group, but this difference was not statistically significant.

**Conclusion:**

After 3 months, there was no significant difference between the *P. atlantica* and control groups in terms of blood sugar and lipid profiles. The mean BP in patients with DM receiving *P. atlantica* oleoresin was significantly reduced compared with that in the beginning of the study. Also, these changes were significant compared with the control group.

## Graphical abstract

Consumption of *Pistacia atlantica* oleoresin by patients with diabetes mellitus can improve the metabolic conditions of these patients in terms of blood sugar and lipid profile. However, this difference was not statistically significant. However, the mean changes in blood pressure before and after the intervention in the intervention group were significantly higher than those in the control group.

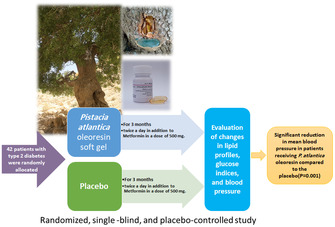

## 1 Introduction

Diabetes mellitus (DM) is the most common endocrine disorder affecting more than 285 million people worldwide. It is expected that this number will reach 438 million people by 2030 [1]. DM is a metabolic disorder with multiple aetiologies, characterised by chronic hyperglycaemia with disturbances in carbohydrate, fat and protein metabolism due to defects in insulin secretion, insulin action or both [2].

Cardiovascular disorders (CVDs) and liver damage are among the long‐term complications of poorly controlled DM [3]. Microvascular complication of DM includes retinopathy, neuropathy and nephropathy, all of which can lead to disability, dependence and mortality [4]. DM is also associated with hypertension (HTN), dyslipidaemia, decreased fibrinolytic activity, severe arteriosclerosis and increased platelet aggregation [1]. HTN and Type 2 DM are mutual metabolic disorders that make a person highly susceptible to atherosclerotic CVDs and kidney failure [2]. With early interventions to control DM and other CVD risk factors, microvascular damage can be reversible before irreversible changes occur [5].

The management of increased blood sugar (BS), blood pressure (BP) and serum lipids with the least side effects is still a very important challenge for the medical system [1]. In addition, the challenge with conventional drugs in the successful treatment of many chronic diseases such as DM, disorders related to CVDs and the desire of people to use complementary and herbal medicines has been a progressive global trend [6]. All over the world, different ethnic groups use their native medicinal plants on the basis of long‐term traditional experiences [7].

*Pistacia atlantica* Desf. is a species of pistachio from the Anacardiaceae family and is found in the Zagros Mountains of Iran (western and northwestern Iran with moderate climate). Oleoresin derived from *P. atlantica*, which is called gum, is used to produce chewing gum. The fruit of *P. atlantica* is consumed as food by the inhabitants, and its unripe fruit is served as a pickle. Its shell and kernel oil are consumed by local people as frying oil [8].

In the sources of Persian traditional medicine, *P. atlantica* oleoresin has been mentioned to have various therapeutic effects such as improving digestive disorders, eliminating inflammation and diuretic effects [9, 10]. On the basis of the theoretical concepts of Persian medicine, *P. atlantica* oleoresin might help to reduce the accumulation of harmful waste substances that contribute to the occurrence of metabolic diseases [9].

*Pistacia atlantica* gum mainly contains monoterpene compounds α‐pinene, β‐pinene, camphene, limonene, α‐terpineol and triterpene such as oleanolic acid (OA), ursonic acid, masticadienonic acid, masticadienolic acid, as well as phenolic compounds, sterol and fatty acid [11].

Various studies have shown the BP‐lowering effects of monoterpene compounds such as α‐pinene [12]. In a study on rats and guinea pigs, α‐pinene, as one of the main components of black seed essential oil, decreased arterial BP and caused bradycardia in a dose‐dependent manner [13, 14]. Also, after the intravenous administration of both α‐pinene and *p*‐cymene in rats, reduction BP level and bradycardia were observed. These effects are probably due to the inhibition of the vasomotor centre, resulting in a decrease in arterial BP and heart rate [15]. In other studies, the effects of cardiovascular protection and BP reduction by monoterpenes in plant essential oils have been mentioned [16, 17].

The lipid‐lowering effect of *Pistacia lentiscus* oleoresin (which is chemically similar to *P. atlantica* oleoresin) was investigated in mice sensitive to detergent‐induced hyperlipidaemia. Administering mastic oleoresin to healthy rats led to a dose‐dependent decrease in the cholesterol and triglycerides synthesis. Also, in mice with high serum lipids, the treatment of mastic oleoresin had a strong hypolipidaemic effect. In terms of constituent compounds, six compounds, β‐caryophyllene, α‐pinene, β‐pinene, camphene, β‐myrcene and linalool, constitute 65%–80% of the total weight of extracted oleoresin. By testing different components of mastic oleoresin, it was shown that serum fat reduction is related to camphene. The treatment of HepG2 cells, a hepatoblastoma cell line, with camphene resulted in a decrease in cellular cholesterol content to the same extent as mevinolin, a known β‐Hydroxy β‐methylglutaryl‐CoA (HMG‐CoA) reductase inhibitor. The lipid‐lowering action of camphene is independent of HMG‐CoA reductase activity, which indicates that its hypocholesterolaemic and hypotriglyceridaemic effects are related to a different mechanism of action from statins [18].

Anti‐inflammatory effects of and reduction in BS with α‐pinene were observed in mice [19]. An absorption of α‐pinene, β‐pinene and myrcene in mastic oil and its protective effect against lipid oxidation have also been observed in humans [20].

OA, another important compound in *P. atlantica* oleoresin, has antihypertensive effects [21]. Also, by administering OA‐rich extract from Alhagi root orally to diabetic rabbits, a significant decrease in BS, lipids and lipid peroxidation (malondialdehyde [MDA]) has been observed [22].

In animal studies, investigating the acute toxicity of *P. atlantica* essential oil has a high safety in rats, which indicates its high safety in consuming this plant [11].

Considering the mentioned properties and also considering that *P. atlantica* is one of the native plants of Iran and the safety of its consumption is mentioned in the articles, it is available and cheap, in the present study it was decided to investigate the efficiency and effectiveness of the oleoresin product of turpentine (*P. atlantica*) in improving BP, BS and serum lipid levels in patients with Type 2 DM in a randomised, placebo‐controlled, clinical trial study.

## 2 Materials and Methods

### 2.1 Oleoresin Collection

Oleoresin was obtained from Kermanshah city and authenticated by an expert botanist (Dr. Abbas Golpoor; Payam Nour University). Voucher specimen (no. BMS‐412) was deposited in the Phytopharmaceutical Laboratory of the Faculty of Traditional Medicine, Babol University of Medical Sciences.

#### 2.1.1 Extracting Essential Oil for Oleoresin Standardisation

In order to standardise the treatment protocol, essential oil of the oleoresin (200 g) was extracted. The extraction of essential oils was made using the hydrodistillation method and by the Clevenger apparatus for 4 h. The obtained essential oil was dehumidified by sodium sulphate (Siga, Germany) and stored at 4°C in a dark vial until analysis via gas chromatography–mass spectrometry in the Jahad Daneshgahi Institute of Medicinal Plants, a professional centre of analysis (Table 1). The system characteristics were as follows: the gas chromatography device: Agilent 6890; column length of 30 m, inner diameter of 0.25 mm; layer thickness of 0.25 μm; Type BPX5. The sample that was diluted by *n*‐hexane was injected into the GC/MS (1 μL). The temperature program of the column was described in a previous study [23]; the temperature of the injection chamber: 290°C, split 1:35; the carrier gas: helium (flow rate of 0.5 mL/min). The mass spectrometer: Agilent 5973; ionisation voltage: 70 eV, EI ionisation method and ionisation source temperature of 220°C. The scanning range of mass: 40–500. The chemstation software was used [24, 25].

**TABLE 1:** Chemical composition of oleoresin [23].

No.	RT	%	Components	KI_C_	KI_R_	Type
1	10.95	0.10	Tricyclene	924	926	MH
2	11.57	87.90	Alpha‐pinene	936	939	MH
3	12.39	0.72	Camphene	952	954	MH
4	13.57	0.43	Sabinene	976	975	MH
5	13.83	2.38	Beta‐pinene	981	979	MH
6	14.41	0.32	Beta‐myrcene	993	991	MH
7	14.89	0.05	2‐Carene	1002	1002	MH
8	15.40	0.51	3‐Carene	1012	1011	MH
9	15.89	0.06	Alpha‐terpinene	1021	1017	MH
10	16.39	0.19	Ortho‐cymene	1031	1026	MH
11	16.54	0.74	Limonene	1034	1029	MH
12	16.73	0.41	Cineole <1,8‐>	1038	1031	MO
13	19.46	1.71	Alpha‐terpinolene	1090	1089	MH
14	20.30	0.11	Linalool	1107	1097	MO
15	22.52	1.03	Bicyclo[2.2.1] hept‐2‐en‐7‐ol	1151	1151	MO
16	22.75	0.96	Trans‐verbenol	1156	1156	MO
17	24.97	0.33	Meta‐cymene‐8‐ol	1201	1180	MO
18	25.27	0.56	Alpha‐terpineol	1207	1189	MO
19	25.96	0.06	d‐Verbenone	1222	1205	MO
20	29.28	0.53	Bornyl acetate	1292	1289	MO
21	31.76	0.09	2‐Oxabicyclo[2.2.2]octan‐6‐ol, 1,3,3‐trimethyl‐, acetate	1348	1348	SO
		99.19	**Total identified**			
			Monoterpene hydrocarbons	MH		88.72
			Oxygenated monoterpenes	MO		0.43
			Sesquiterpene hydrocarbons	SH		0.43
			Oxygenated sesquiterpenes	SO		2.81

#### 2.1.2 Soft‐Gel Preparation

Oleoresin fill material was prepared by a pharmacist in the Phytopharmaceutical Laboratory of the Faculty of Traditional Medicine, Babol University of Medical Sciences. The softgel was prepared by Barij Essan Pharmaceutical Company. Softgel capsule (500 mg) containing 350 mg of *P. atlantica* oleoresin in 150 mg of base oil (vegetable oil) was used in the intervention group. Experiments related to the control of microbial contamination of drugs were carried out in the Jahad Daneshgahi Institute of Medicinal Plants, a professional centre of analysis. The microbial limit tests were performed in accordance with the USP43 for herbal preparations in Jahad Daneshgahi Institute of Medicinal Plants, a professional centre of analysis. The results of the microbial tests were in accordance with the standard limits.

### 2.2 Study Design

#### 2.2.1 Sample Collection

This study is a randomised, single‐blind, placebo‐controlled clinical trial conducted among 65 subjects with Type 2 DM that 42 subjects were selected for the study. Patients with Type 2 DM were recruited from the specialised and sub‐specialised clinic of Deziani, which is affiliated to the Golestan University of Medical Sciences, Gorgan, Iran. We divided patients into the two groups, namely, intervention and control groups.

### 2.3 Participants

From March 2022 to July 2023, 65 patients were enrolled in the study, but only 42 of them met the inclusion criteria and were divided into two groups: control and intervention, 21 patients in each group. In this study, samples were selected as simple random sample method, then the samples for the study groups were randomly allocated using permuted block design.

#### 2.3.1 Inclusion Criteria

Patients with Type 2 DM (fasting blood glucose >126), aged 30 and 64 old, history of having clinical diagnosis of Type 2 DM for <2 years, taking at least one oral hypoglycemic drugs such as metformin for initial control, HbA1c ≥8 and ≤10.

#### 2.3.2 Lost to Follow‐Up Criteria

The lost to follow‐up criteria are as follows: allergy to *P. atlantica* oleoresin, increase in blood glucose level during study, complication of drug.

### 2.4 Study Design

In this single‐blind trial study, 42 patients were recruited using the randomised permutation block design (blocks of 4) with random permutations (AABB, ABAB, ABBA, BAAB, BABA, and BBAA). In the intervention group, 500‐mg *P. atlantica* oleoresin capsules were administered twice a day for three consecutive months of trial in addition to 500‐mg metformin. Meanwhile in the control group, 500‐mg metformin was the only medicine that was used for 3 months of trial. Before the beginning of study and 3 months after that, FBS, HbA1c, total Chol, LDL, high‐density lipoprotein (HDL) and BP were measured for both groups and were compared with each other.

### 2.5 Sample Size

**FIGURE 1:**
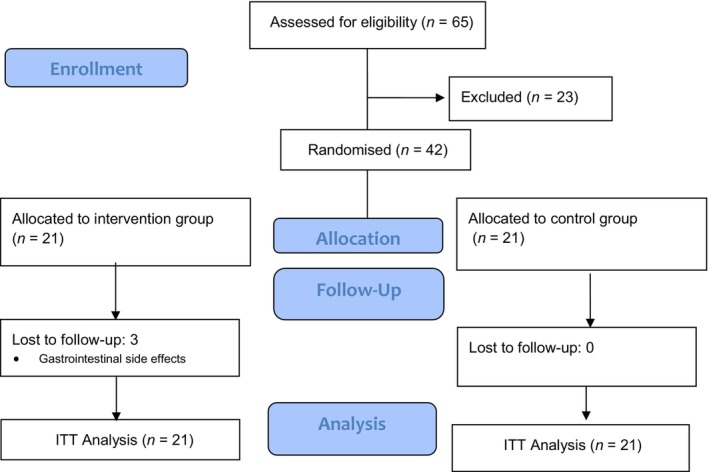
Consolidated Standards of Reporting Trials (CONSORT) follow chart of the clinical study.

According to the results of the previous study, the changes in mean ± standard deviation (SD) for serum blood sugar, LDL and HDL variable based on the confidence interval of 95%, and power of 80%, were considered to calculate sample size [26]. The number of patients computed per group was 21. After recruiting 65 patients, only 42 of them met the inclusion criteria. A diagram of the study design is shown in Figure 1.

### 2.6 Recording Possible Adverse Effects of the Medications

During the course of treatment, the drugs were delivered to the participants every 4 weeks, but each week they were checked for medicine consumption and possible unwanted side effects of the drug.

### 2.7 Statistical Analysis

Statistical analysis was performed using SPSS, version 16. Quantitative data were described as mean and SD. However, qualitative data were described through frequency distribution table. The normality distribution of quantitative data was evaluated using the Shapiro–Wilk test. Mean values were compared using the independent *t*‐test and the Mann–Whitney *U* test. *p* value <0.05 is considered significant.

## 3 Results

### 3.1 GC–MS Analysis of *P. atlantica* Essential Oil

The GC/MS analysis of the essential oil exhibited that among identified compounds with amount percentage more than 1%, α‐pinene was the major component (87.9%) of the essential oil composition (Table 1).

### 3.2 Baseline Characteristics of the Participants

In this study, 42 patients with DM participated, 21 patients each in the intervention and control groups. The mean age of the intervention and control groups was 54/66 ± 6/97 and 56/04 ± 11/97 years, respectively. The intervention group had diabetes for a mean of 4 years, whereas the control group had diabetes for a mean of 5 years. The mean body mass index in the intervention and control groups was 28.69 ± 3.38 and 29.95 ± 8.58, respectively. There were no considerable differences between the two groups' baseline characteristics of the demographic characteristics (Table 2). The laboratory variables of blood sugar control and lipid profile were measured between the two groups before the intervention. The results showed that both the intervention and control groups were homogeneous in terms of these variables (Table 3).

**TABLE 2:** Comparison of demographic characteristics between the intervention and control groups.

Variable	Group	Mean ± SD	*p*
Age	Intervention	54.66 ± 6.96	0.28
	Control	56.04 ± 11.79	
Duration of diabetes	Intervention	4.90 ± 3.59	0.57
	Control	5.38 ± 3.35	
BMI	Intervention	28.69 ± 3.38	0.82
	Control	8.58 ± 29.95	

**TABLE 3:** Comparison of the mean indicators of blood sugar control and lipid profile and blood pressure between the intervention and control groups.

Variable	Group	Before intervention		After 3 months		Mean of difference	
		Mean ± SD	*p*	Mean ± SD	*p*	Mean ± SD	*p*
FBS	Intervention	198 ± 73.95	0.51	154.09 ± 34.14	0.18	−43.90 ± 66.96	0.88
	Control	180.29 ± 53.48		141.81 ± 43.40		−38.47 ± 49.29	
BS	Intervention	291.57 ± 70.66	0.30	241.10 ± 68.35	0.46	−50.47 ± 77.50	0.65
	Control	264.62 ± 93.05		225.19 ± 69.11		−39.42 ± 81.53	
HbA1C	Intervention	8.58 ± 1.52	0.87	7.96 ± 1.15	0.95	−0.62 ± 1.36	0.89
	Control	8.50 ± 1.76		7.94 ± 1.42		−0.56 ± 1.39	
Total Chol	Intervention	179.19 ± 38.15	0.75	161.24 ± 28.16	0.90	−17.95 ± 49.79	0.74
	Control	175.09 ± 46.10		162.48 ± 33.31		−12.62 ± 52.47	
HDL	Intervention	44.95 ± 9.27	0.26	43.19 ± 7.45	0.32	−1.76 ± 8.50	0.86
	Control	41.71 ± 9.07		40.76 ± 7.39		−0.95 ± 10.71	
LDL	Intervention	102.95 ± 34.45	0.75	80.90 ± 16.53	0.94	−22.04 ± 33.49	0.84
	Control	99.86 ± 28.65		87.48 ± 33.38		−12.38 ± 21.25	
TG	Intervention	170.52 ± 75.73	0.76	144.52 ± 49.37	0.91	−26 ± 87.53	0.25
	Control	159.67 ± 67.15		162.95 ± 91.82		3.28 ± 76.12	
Bp	Intervention	129.81 ± 18.60	0.048	118.39 ± 8.36	0.44	−11.41 ± 15.48	≤0.001
	Control	118.83 ± 10.85		123.09 ± 14.77		4.26 ± 11.18	

### 3.3 Assessments of Outcomes

At the end of the study, 39 patients accomplished the research, and three patients were left out because of gastrointestinal complications. Missing data were replaced by standard technique for intention‐to‐treat (ITT) analysis (Figure 1). The comparison of the mean laboratory variables of blood sugar control and lipid profiles between the two groups after the intervention showed that the mean of total cholesterol, LDL and TG in the intervention group was lower than that in the control group, but this difference was not statistically significant (Table 3).

The results of comparison of mean changes in BS control indicators and lipid profile before and after the intervention by the intervention and control groups showed that the mean change in total cholesterol, LDL and HDL in the intervention group was higher than in the control group. It means that the intervention had reduced the mean of total cholesterol, LDL and HDL, but these differences were not statistically significant, and the BP variable in the intervention group showed a significant decrease (Table 3).

## 4 Discussion

Diabetes is a chronic metabolic disorder characterised by high blood sugar levels due to impaired insulin production or insulin resistance. It is a global health concern with significant implications for morbidity and mortality [27]. There is practical importance of different medicinal herbs such as *Petroselinum crispum* [28], peppermint [29] and many other edible plants in the management of chronic diseases [30]. Persian medicine has long‐utilised natural remedies [31], such as *P. atlantica*, for various diseases. In this randomised clinical trial, the effects of *P. atlantica* oleoresin in improving BP, BS and serum lipid levels were assessed in patients with Type 2 DM.

A number of preclinical studies [3, 27] have investigated the impact of *P. atlantica* on diabetic condition, in vivo. The oleoresin effect (200 mg/kg) of the plant was studied by Bagheri et al. [3] in diabetic rats. The study found that *P. atlantica* oleoresin treatment significantly decreased MDA and increased GSH, GPx, CAT and SOD levels in diabetic rats. The results suggest that *P. atlantica* could be a potential agent for protecting against diseases associated with oxidative stress [3]. The results of our study showed that there was no significant difference between the *P. atlantica* and control groups in terms of blood sugar and lipid profiles. However, the mean changes in BP before and after the intervention in the intervention group were significantly more than those in the control group.

A triple‐blind randomised placebo‐controlled study on subjects with diabetic gastroparesis evaluated the effect of *P. atlantica* gum (2 g; two times a day, for 1 month) on their symptoms. According to the results, after an 8‐week intervention, *P. atlantica* gum significantly reduced the severity of gastroparesis symptoms as well as BMI, and HbA1c [32]. This different effect might be due to the higher dose of the oleoresin than that of our study. These effects can be attributed to the main compound of its essential oil, alpha‐pinene, which has been evaluated for its influence on blood glucose and lipid levels, in vivo. Alpha‐pinene (100, 200 mg/kg, daily for 14 days) was shown with a significant decreasing effect on glucose, triglyceride, total cholesterol and low‐density lipoprotein levels in diabetic rats. The antioxidant properties of alpha‐pinene suggest its potential to treat diabetes [33]. beta‐Pinene, the other major phytochemical of *P. atlantica*, was also shown to have hypoglycaemic and hypolipidaemic effects in alloxan‐induced diabetic rats possibly due to its anti‐inflammatory effects, which was evaluated via the carrageenan‐induced models [34]. The phytochemical analysis in our study also showed that α‐pinene was the major component (87.9%) of the essential oil composition. Moreover, beta‐pinene (2.38%) was the other main compound identified (Table 1). Similar to our study, in the research by Mahjoub et al. [32], the systolic BP (*p* = 0.006) significantly decreased in the *P. atlantica* group after the treatment.

There are also other clinical studies that evaluated the effect of the different parts of *P. atlantica*, rather than its oleoresin, on participants with diabetes. In a double‐blind, placebo‐controlled clinical trial, patients with Type 2 DM received *P. atlantica* (10 cc of distilled water two times a day) for 3 months. There was a significant decrease in FBS in the intervention group compared with the placebo [35]. Moreover, a randomised, triple‐blind, placebo‐controlled study compared *P. atlantica* kurdica fruits and placebo capsules for patients with Type 2 DM with hyperlipidaemia. Results showed significant reductions in 2HPP, total cholesterol and LDL‐c after 1 and 2 months of the intervention, whereas there were not any significant alterations in FBS, HbA1c, TG, HDL‐c, ALT, AST and Cr. The herbal capsule was standardised according to benzoic acid, rutin and quercetin standards, with α‐pinene as a major volatile constituent [36]. The differences in these studies' findings might be due to the difference in the form of the administered *P. atlantica* preparations.

*Pistacia atlantica* and *P. lentiscus* (Mastic) oleoresins are very similar in chemical composition. And in traditional Persian medicine, they can be used as substitutes for each other.

Animal studies confirm the effect of reducing systemic arterial BP by *P. lentiscus* oleoresin in rats [37, 38]. Tzani et al. [37] studied the effects of mastic on high BP in rats (40 mg/kg of body weight per day of mastic for 2 weeks). Mastic reduced the damage of the target organ by improving the biomechanical properties of the aorta, and returning the thickness of the hypertrophy of the small vessels of the myocardium. The protection of mastic against high BP was determined by maintaining the serum albumin level. Mastic also led to a decrease in CRP and IL‐6 levels. The results showed that mastic has the effect of reducing BP through the downregulation of renin secretion related to the reduction in target organ damage and inflammatory conditions [37]. In a randomised, double‐blind, controlled crossover clinical study, Kontogiannis et al. [39] studied the effects of Chios mastic on peripheral and aortic haemodynamics and related changes in the gene expression of molecules related to hypertension. On the basis of the results, oral consumption of 2800 mg (four 700‐mg tablets) of mastic by hypertensive patients led to a decrease in peripheral and aortic systolic BP and peripheral pulse pressure. Gene expression analysis indicated the downregulation of proteasome system and NOX2 pathway [39]. Compared with our study, the prescribed dose in their study is much higher and the BP‐lowering effects have been revealed more clearly.

## 5 Limitations

Because all participants, especially the patients with chronic diseases, had required ongoing medical contacts, the potential limitation is lost to follow‐up patients based on their health status. It is unclear whether the findings would be replicated with other specific populations or with a large sample.

## 6 Conclusion

In this randomised clinical trial, the effects of *P. atlantica* oleoresin in improving BP, BS and serum lipid levels were assessed in patients with Type 2 DM. Our study's results showed no significant difference between the *P. atlantica* and control groups in terms of blood sugar and lipid profiles. However, the mean changes in BP in the intervention group after the intervention were significantly more than that of the control group. *P. atlantica* capsule seems to have the potential to be further studied as a supplement to lower BP in diabetics. Considering that it is possible to use higher doses of oleoresin, the effects of reducing BP and improving sugar and lipid profiles may be more evident in higher doses of the drug. Therefore, placebo‐controlled studies with a higher sample size and different drug doses are suggested.

## Author Contributions

**Zahra Memariani:** conceptualization (lead), project administration (equal), writing–review and editing (equal). **Mahin Tatari:** formal analysis (equal), writing–review and editing (equal). **Maryam Zahedi:** data curation (equal). **Zahra Hesari:** writing–review and editing. **Ali Davarian:** data curation (equal). **Fatemeh Kolangi:** conceptualization (lead), project administration (equal), writing–review and editing (equal).

## Ethics Statement

This study was reported in compliance with the recommendations of the Consolidated Standards of Reporting Trials (CONSORT) 2010 statement and adhered to the principles of the Declaration of Helsinki. All patients signed the written informed consent. The code of ethics of the present study was approved by the Golestan University of Medical Sciences (IR.GOUMS.REC.1400.449) and then registered in the Clinical Trial Registration Number: IRCT20161031030616N2 in the clinical trials registry of Iran. Moreover, all methods were performed in accordance with the relevant guidelines and regulations.

## Conflicts of Interest

The authors declare no conflicts of interest.

## Data Availability

The data that support the findings of this study are available from the corresponding author upon reasonable request.
